# The impact of collaborative pharmaceutical care on hospital discharge medication error prevalence: A stepped-wedge cluster randomised trial

**DOI:** 10.1016/j.rcsop.2025.100638

**Published:** 2025-07-31

**Authors:** Gráinne Kirwan, Ann Allen, Evelyn Deasy, Tim Delaney, Jennifer Hayde, Ciara McManamly, Catherine Wall, John O'Byrne, Tamasine Grimes

**Affiliations:** a School of Pharmacy and Pharmaceutical Sciences, Trinity College Dublin, Dublin, Ireland; b Pharmacy Department, Tallaght University Hospital, Dublin, Ireland; c Management Team and Medical Directorate, Tallaght University Hospital, Dublin, Ireland; d Pharmacy Department, The National Rehabilitation Hospital, Dublin, Ireland

**Keywords:** MeSH: Pharmaceutical care, Transition of care, Medication reconciliation, Medication error, Patient safety

## Abstract

**Objectives:**

The benefits of a collaborative approach to medication management between pharmacists and clinicians in secondary care on patient safety has been demonstrated in clinical trials. However, less is known about the benefit of such collaboration in real-world settings. This study assessed the effectiveness of a collaborative model of pharmaceutical care including pharmacist collaborative prescribing, on discharge medication error, and explored the intervention fidelity.

**Methods:**

This stepped wedge cluster-randomised controlled trial was undertaken at a university hospital in Dublin, Ireland. A cluster was one or more medical or surgical specialty, or part thereof, delivering acute care. Adult patients, using five plus regular medicines pre-admission, receiving care from a participating cluster, and discharged alive from that cluster were eligible for inclusion. Patients previously admitted during the study period and enrolled were excluded. The intervention saw a pharmacist aligned to a specialty, delivering collaborative services to patients: medication history taking, admission medication reconciliation, inpatient medication optimisation, discharge medication reconciliation and collaborative prescribing. The comparator was ward-based pharmacist care. Sample size accounting for study design, attrition and effect size in discharge medication error was calculated as 430 participants. The primary analysis was undertaken by the intention-to-treat (ITT) principle and multilevel logistic regression through the Generalised Linear Mixed Model (GLMM) procedure, was used to account for the effect of clustering and adjust for confounders.

**Results:**

Eighty-six of 432 (19.9 %) assessable patients experienced a clinically significant discharge medication error, 37 (43 %) of whom were intervention group patients. Intention-to-treat analysis suggested no difference in the likelihood of experiencing this primary outcome between study groups (adjusted odds ratio 1.24, 95 % confidence interval 0.53–2.88). This finding was consistent in the extreme sensitivity and per protocol analyses. Intervention fidelity was poor with six (2.4 %) intervention patients receiving discharge medication reconciliation.

**Conclusion:**

Under real-world conditions, this collaborative model of pharmaceutical care including medication reconciliation and collaborative prescribing was equivalent to standard care in protecting against clinically significant discharge medication error. Future research should employ an implementation science framework to better understand how pharmaceutical care at discharge can be spread.

## 1 Introduction

Medication optimisation to enhance patient safety and other outcomes remains a current global priority. Hospital inpatient clinical pharmacy is an essential aspect of the medication optimisation journey for any patient experiencing hospitalisation. Previous research suggests the benefits of a collaborative approach between hospital pharmacists and clinicians and of focussing on transitions into and out of hospital (WHO TOC).^1^ Collaborative pharmaceutical care refers to the delivery of care by pharmacists in an integrated manner, collaborating with other members of the healthcare team. However, several issues limit the development of collaborative pharmaceutical care models such as: (1) the absence of clinical pharmacy practice guidance globally or nationally beyond the United Kingdom (UK), the United States of America (USA), Canada and Australia,^2^ (2) a disconnect between policy and practice with studies identifying a continued high prevalence of medication error at care transitions in jurisdictions with relevant policy, e.g. the UK,^3^ and those without, e.g. Norway,^4^ (3) the dearth of evidence-based, validated or feasible key performance indicators for clinical pharmacy services^5^^,^^6^ and (4) advocacy for multidisciplinary working in many of the available clinical pharmacy guidelines, but limited detail about how to deliver this in practice.^2^

A collaborative model of pharmaceutical care incorporating pharmacist-led medication reconciliation and collaborative prescribing was developed at a university hospital in Dublin, Ireland.^7^ Collaborative pharmaceutical care involved the pharmacist being aligned with a medical or surgical team, leading on medication reconciliation at care transitions and liaising closely with the team to discuss issues arising throughout the inpatient journey and making major or minor changes to the prescription chart (collaborative prescribing). An uncontrolled before-after study of the model, published in 2014, suggested that this complex intervention was associated with a reduced prevalence of medication error at hospital discharge.^7^ An associated cost-utility analysis suggested that the model was likely to be cost-effective. The evidence of (cost-)effectiveness supported the model's implementation within the study hospital on a wider scale, replacing the standard pharmacy services.^8^^,^^9^ This study was undertaken in parallel with the roll-out of the reconfigured clinical pharmacy service.

It was necessary to determine whether the real-world implementation of the service model on a wider scale, which was associated with significant organisational change, had a positive impact, consistent with the original study. Demonstration of a consistent, sustained effect in the context of real-world practice could be of interest to policy makers and other healthcare organisations considering the introduction of a similar service. The evidence would be valuable to support efforts to establish clinical pharmacy guidance and standards, key performance indicators and to support continuous quality improvement. ^2^^,^^5^^,^^6^ It would also provide further evidence on the implementation of a model of collaborative pharmaceutical care to complement existing evidence predominantly from the UK and USA.^10^ The aim of this study was to establish the effectiveness of a collaborative model of pharmaceutical care in reducing discharge medication error, following its adoption by medical and surgical specialties in a university hospital, and to explore the fidelity to the intended model of service.

## 2 Materials and methods

### 2.1 Study setting and design

The stepped wedge cluster randomised trial (SW-CRT) was undertaken at an acute university hospital: a tertiary hospital located in suburban Dublin, managing >18,000 inpatient episodes per year and providing care to a catchment area of 0.5 million people living in a mixture of affluent and deprived areas. The standard pharmaceutical care in the study hospital is described below (Section 2.4).

A stepped wedge design was chosen because the previously piloted intervention was being implemented into routine care in a real-world setting.^11^ Guidance for reporting SW-CRTs was followed.^12^ A cluster was defined as one or more medical or surgical specialties, or part of a single specialty, delivering care to adult inpatients. The composition of the eight clusters was determined by routine patient volume in the ten component specialties. The clusters were the units of randomisation.

**Fig. 1:**
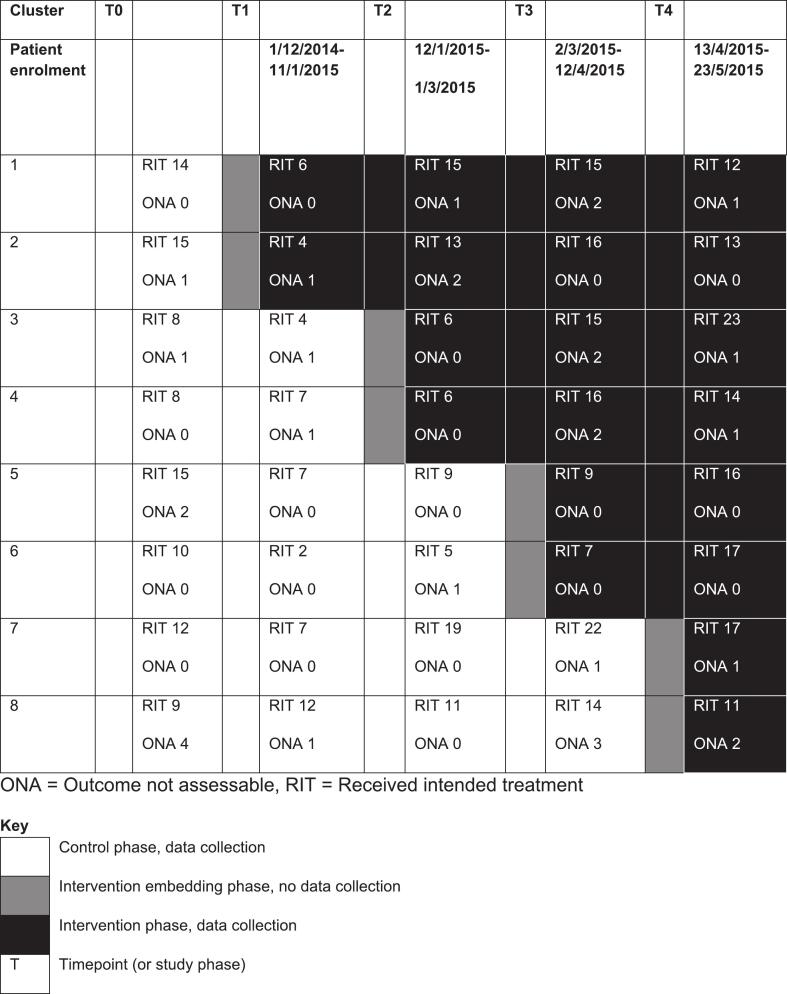
Schematic of stepped wedge study design.

Clusters were randomised in blocks, by a pharmacist who was independent of the study team, using the random number generator in Microsoft Excel®, to one of four intervention start dates and crossed over sequentially in random order from the control (standard care) period to the intervention period until all clusters were exposed to the intervention (Fig. 1).^13^ The same intervention was delivered across all clusters. Two clusters switched from standard to intervention care at each timepoint. A baseline data collection period across all clusters preceded introduction of the intervention to the first two clusters. Upon intervention initiation in each cluster, patient enrolment was suspended for two-weeks to allow the intervention to embed within that cluster. Thereafter, data collection recommenced. Cross-sectional outcome measurements were collected, as described in Section 2.5.^14^ Patients were recruited between October 2014 and May 2015 and data were collected until July 2015.

### 2.2 Participants

Patients aged 18 years or older, using at least five regular medicines before admission, who received acute care from a participating cluster in either the standard- or intervention- study group, and discharged alive from that cluster were eligible for inclusion. Patients previously admitted during the study period and enrolled were excluded. The unit of observation was patients within the cluster. Random samples of patients admitted in a participating cluster and who met the inclusion criteria were recruited.

Pharmacists in both study groups delivered care to patients in order of clinical need, consistent with contemporary standard practice. Prioritisation was based on patient age, number of pre-admission medications, comorbidities, use of high-risk medication and renal function. It was possible that recruited patients received no, some or all components of the service model. An overview of the features of the intervention and comparator are presented (Table 1) and elaborated upon below.

**Table 1:** Description of standard care and intervention.

Feature	Standard care	Intervention
Service arrangement	Aligned to a ward	Aligned to a specialty
Pharmacists involved were the same pool across both study groups	Had met local minimum standards to deliver a clinical pharmacy service. (Intervention pharmacists not yet deployed to the intervention phase delivered standard care)	Three plus years post registration experience, Postgraduate qualification in clinical pharmacy.
Service at admission		
*Medication history taking*	Yes	Yes
*Use electronic MedRec tool*	Yes	Yes
*Admission medication reconciliation*	Yes, recorded in the healthcare notes and needed communication with and action by physicians or surgeons to resolve identified discrepancies	Yes, led by intervention pharmacist with collaborative prescribing (see below) which enabled the pharmacist to address and resolve discrepancies by making changes to the medication prescription and administration record and to record the actions taken in the healthcare notes
Service during admission		
*Prescription review and endorsement*	Yes	Yes
*Routine clinical tasks*	Yes	Yes
*Independent edits of drug order*	Yes, Instigated by pharmacist via request to physicians or surgeons	Yes, changes could be made by pharmacist directly to medication prescription and administration record and team informed verbally/via notes
*Collaborative prescribing*	No	Yes
Service at discharge		
*Discharge medication reconciliation*	Not routinely, although pharmacists could intervene as they deemed necessary or if it was requested by the medical/surgical team	Yes, with an aim to deliver to all patients
*Major and minor edits of drug order*	No	Yes
*Collaborative prescribing of discharge prescription co-signed by a medical practitioner*	No	Yes

PAML = pre-admission medication list.

### 2.3 Intervention

The intervention was delivered by pharmacists to patients under the care of consultant physicians and surgeons within the participating specialties assigned to each cluster. A service level agreement between the Pharmacy Department and the physicians or surgeons detailed the nature of the intervention to be delivered. The pharmacist was empowered to collaboratively prescribe and could amend the inpatient drug prescription chart, where necessary, and could contribute to generating the electronic discharge medication list, reconcile it with the pre-admission medication list (PAML) and any subsequent modifications or additions made during the inpatient stay.

### 2.4 Comparator

Standard care was delivered by pharmacists aligned to a medical or surgical ward. Patients on the pharmacist's ward who were under the care of a consultant physician or surgeon within the intervention phase were seen by intervention pharmacists.

### 2.5 Outcome measures

The proportion of patients with at least one clinically significant medication error at discharge, identified through independent medication reconciliation by the lead investigator (a pharmacist), was the primary outcome measure. This was defined as an error with a mean potential severity score ≥ 5 on a validated, reliable visual analogue scale running from 0 to 10 (0 = no harm – 10 = death), as determined by averaging the scores returned by four independent assessors who were blinded to study group and who reviewed a written case vignette describing the case and the error.^15^ These clinical significance assessors were hospital pharmacists, community pharmacists, hospital doctors or general practitioners, and this method has been used by the authors in multiple previous studies.^7^^,^^16^^,^^17^ Where a patient experienced multiple errors, the error with the assessors' highest mean score was used. Medication error was validated by two pharmacists independent of intervention or comparator service delivery.

Secondary outcome measures were the proportion of patients with one or more medication errors of any potential severity at discharge, and intervention fidelity, as captured from the presence or absence of pharmacist documentation in the healthcare record describing their input.

### 2.6 Sample size

The sample size calculation was based on the primary outcome measure. The previous study findings suggested that the intervention reduced the prevalence of the primary outcome from 15.8 % to 0.9 % of patients.^7^ To demonstrate a similar reduction with 80 % power and a significance level (two-sided) of 5 %, 106 patients were required for an individually patient randomised controlled trial of two parallel groups. The intraclass correlation coefficient (ICC) was estimated as 0.04. The design effect was calculated as 3.36, assuming a cluster size of 60 patient participants. Multiplying the design effect by the number of participants per treatment arm in an individually randomised trial resulted in a sample size of 358, which was conservatively inflated by 20 % to account for attrition resulting in a final total of 430 patients.

### 2.7 Recruitment and data collection

Patient participants were identified through the hospital administrative system. A list of newly admitted patients to each participating cluster was created daily and sorted in random order on a Microsoft Excel spreadsheet using the RAND() function and, of those, patients fulfilling the inclusion criteria were enrolled sequentially into the study. The clusters were randomised to transition from usual care to the intervention at one of four timepoints as described in Section 2.1. The same intervention was delivered across all clusters. The trial was stopped when all clusters were randomised and received the intervention for the required duration. Demographic (age, gender), comorbidity and process data were collected from the healthcare record and classified by study group. Process data included whether the patient's discharge prescription was handwritten or electronically generated, and the pharmacist's input to care. Where the PAML was already documented by a clinical pharmacist, these data were utilised by the investigator. Otherwise, the investigator constructed the PAML, using a method previously described.^7^ Data pertaining to outcome measures were collected by an independent pharmacist investigator at the time of patient discharge, with recourse to primary care providers, where necessary, to obtain copies of hospital discharge prescriptions. The discharge prescription was reconciled against the PAML along with any changes made throughout the admission to identify discrepancies. Discrepancies were verified as intentional or unintentional by reference to the clinical pharmacist or treating team.

### 2.8 Blinding

Outcome measures were assessed blindly. The presence of error was retrospectively validated independently by two pharmacists blinded to study arm. The validated errors were assessed for potential severity by a panel of four assessors blinded to study arm. The consultant, other members of the medical or surgical teams, or the clinical pharmacist were not informed which patients were recruited to the study in either study group.

### 2.9 Data management and statistical analysis

Anonymised data were entered into an IBM SPSS® Version 21 database. Double data entry was performed by a second investigator for a random sample of 10 % of patients and checked against the primary entry to ensure consistency. The primary analysis was undertaken by the intention-to-treat (ITT) principle, although both ITT and per-protocol (PP) are reported. Baseline patient characteristics were compared between study groups using standard tests. For the ITT analysis, multiple imputation was employed to replace missing primary outcome measure data and discharge prescription format (handwritten or electronic) data with imputed values. The number of imputations was set at five.^18^

Multilevel logistic regression, through the Generalised Linear Mixed Model (GLMM) procedure, was used to account for the effect of clustering and adjust for confounders. A two-level model was constructed to examine the likelihood of occurrence of the primary outcome measure as a binary response variable. The data set consisted of individual patients (level 1) nested within clusters (level 2). As the outcome measure was dichotomous, a binomial probability distribution and logit link function were selected during GLMM specification. The presence of clustering was determined by estimating the level-2 (cluster) variance for the intercept in the null model and testing for significance through the z-test. Where clustering was not deemed statistically significant, the Generalised Linear Model procedure was employed, specifying a binomial logistic regression model. The variables which were adjusted for were: patient enrolment timepoint, pharmacist input, age, and number of medicines. The presence of pharmacist input was postulated to have an impact on the primary outcome measure, based on previous evidence.^19^ The number of medicines at discharge, inclusive of pre-admission medicines active at discharge and drugs added to a patient's regime during the inpatient episode, were adjusted for, because previous evidence identifies increasing medication burden as a risk factor for discharge medication error.^7^^,^^16^ The independent variables were tested for multicollinearity. A tolerance value of less than 0.20 was considered to indicate likely multicollinearity, while a tolerance statistic of less than 0.10 indicated certain multicollinearity.^20^ As the proportion of clusters exposed to the intervention increases over time, unexposed observations tend to be from an earlier time, resulting in the potential for confounding. Cluster enrolment time was adjusted for by including it as a fixed effect.^13^^,^^14^ The effect of the intervention was reported as an adjusted odds ratio with a 95 % confidence interval.

### 2.10 Appraisal of methods

The study was appraised against the Consolidated Standards of Reporting Trials 2025 statement (retrospectively), its extensions to cluster randomised trials and pragmatic trials^21^, ^22^, ^23^ and its proposed extension to SW-CRT. ^14^ The revised Cochrane risk-of-bias tool for cluster-randomised trials (RoB 2 CRT) was also employed to appraise the study design.^24^

### 2.11 Patient and public involvement

Pharmacy, medical and surgical stakeholders were involved in the discussion about service level agreements and in all operational aspects of the intervention rollout. This research was an active academic practice collaboration, facilitating public involvement at all stages, but it did not involve patients.

### 2.12 Ethical considerations

This study was approved by the institution's Research Ethics Committee (reference 2014–12 Chairman's action (6)). Data were collected by study investigators and were de-identified at entry to the study database and subsequently irreversibly anonymised. As this was a service evaluation, patient consent was not required and data management and confidentiality complied with all legal and policy requirements. It is typical, both in the study setting, and in Ireland generally, that all staff contributing to the clinical care of a patient have access to the patient's healthcare record and can see entries made by other healthcare staff regarding the care of that patient. All intervention pharmacists were staff in the hospital and therefore had routine access to the patient's healthcare record for the purpose of the pharmaceutical services provided.

Any identified clinically significant medication errors were managed in accordance with ethical approval and as undertaken in multiple previous studies by this group: ^7 16^ The error was communicated by the primary investigator to the relevant medical or surgical team, who then took action to follow-up with the patient, their general practitioner or their community pharmacist, as necessary, to facilitate remedial action.

## 3 Results

### 3.1 Description of patient population

**Fig. 2:**
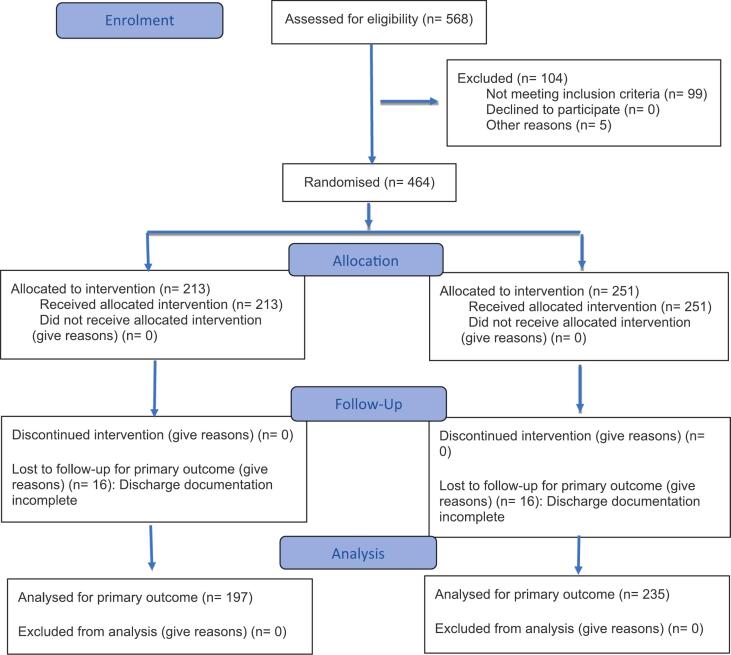
Participant flow diagram.

Four-hundred-and-sixty-four patients (213 control; 251 intervention) met the inclusion criteria and were included in the study. The primary outcome was assessable for 432 patients (Fig. 2). There were no significant differences in the baseline characteristics between study groups (Supplementary material).

### 3.2 Findings

#### 3.2.1 Primary outcome measure

##### 3.2.1.1 Intention-to-treat analysis

Following multiple imputation of missing outcome data for 32 participants, the unadjusted regression output suggested a beneficial effect of the intervention (*p* < 0.01, OR 0.602, (95 % CI 0.375–0.965). After adjusting for covariates (age, gender, number of medicines, phase of study, pharmacist involvement), there was no difference between the groups (Table 2).

**Table 2:** Impact of the study group on the outcome measures.

Outcome	Prevalence		Unadjusted OR (95 % CI; p-value)	Adjusted⁎ OR (95 % CI; *p*-value)
	Standard (*N* = 213) n (%)	Intervention *N* = 251, n (%)		
**Clinically significant DME**	49 of 197 (23.0 %)	37 of 235 (14.7 %)	–	–
Intention-to-treat model	–	–	0.602 (0.375, 0.965; 0.035)¥	1.146 (0.512, 2.565; 0.738)
Per protocol model	–	–	0.564 (0.350, 0.909; 0.019)¥	1.162 (0.551, 2.454; 0.693)
**Any DME**	111 of 197 (56.3 %)	83 of 235 (35.3 %)	–	–
Intention-to-treat model	–	–	0.455 (0.310,0.668; NS)	0.714 (0.385, 1.327; NS)
Per protocol model	–	–	0.447 (0.304, 0.658; <0.001)¥	0.700 (0.378, 1.295; NS)

⁎ adjusted for pharmacist input, study phase, age, gender, and number of medicines.

¥ statistically significant.

##### 3.2.1.2 Per protocol analysis

Eighty-six of 432 patients (19.9 %) experienced a clinically significant discharge medication error, affecting 37 (14.7 %) intervention and 49 (23.0 %) control patients, and the unadjusted regression output suggested a beneficial effect of the intervention (*p* < 0.05, odds ratio (OR) 0.564, (95 % CI 0.350–0.909). After adjusting for covariates (age, gender, number of medicines, phase of study, pharmacist involvement), this effect became non-statistically significant, suggesting there was no difference between the groups (Table 2).

Two independent variables achieved statistical significance in the adjusted logistic regression per protocol and intention-to-treat models: presence of pharmacist input (versus not), and lower number of discharge medicines were protective against experiencing a clinically significant discharge medication error (Supplemental material).

#### 3.2.2 Secondary outcome measures

In the per protocol analysis, 111 of 197 (56.3 %) of standard care and 83 of 235 (35.3 %) of intervention participants experienced one or more discharge medication error of any potential severity, with a median of 2 errors per patient (IQR 1–4), suggesting a beneficial effect of the intervention in the unadjusted model (*p* < 0.001, OR 0.447, 95 % CI 0.304–0.658). However, this became non-significant after adjustment for co-variates (Table 2).

In the intention-to-treat analysis, there was no difference between the groups (Table 2).

#### 3.2.3 Intervention fidelity

Most patients across both study groups received some pharmacist input during the admission episode (Total 344/464, 73.7 %, intervention group 216 (86.1 %), standard care 128 (60.1 %) (p < 0.001, Pearson's chi-square = 40.505) (Table 3). Patients in the intervention group were more likely to receive admission side pharmaceutical care than standard care patients, but there was no difference between the groups in terms of discharge pharmaceutical care, which was low in both groups.

**Table 3:** Intervention fidelity described as proportion of patients who received pharmacist input, by type of activity.

Activity	Standard care (n = 213), n (%)	Intervention (n = 251), n (%)	*p*-value, Pearson's chi-square
Any pharmacist input	128 (60.1)	216 (86.1)	0.000, 40.505
Admission history taking	115 (54.0)	209 (83.3)	0.000, 46.874
Review of inpatient drug administration chart	124 (58.2)	212 (84.8)⁎	0.000, 41.696
Discharge planning or reconciliation	2 (0.9)	6 (2.4)	0.203, 1.433
Medicines information provision	17 (8.0)	32 (12.7)	0.064, 2.773
Patient counselling on medication use	17 (8.0)	14 (5.6)	0.198, 1.068
Suggestion to team to optimise medication use	94 (44.1)	142 (56.6)	0.005, 7.137
Collaborative prescribing	–	99 (39)	–

⁎ denominator = 250 as presence of activity not assessable for one patient.

#### 3.2.4 Risk of bias

The risk of bias assessment (Cochrane RoB2) identified low risk arising from: the randomisation process and selection of the reported result. It identified some concerns due to deviation from the intended intervention, missing outcome data, and measurement of outcome data (Supplementary material).

## 4 Discussion

### 4.1 Key findings

The key findings of this stepped wedge cluster-randomised controlled trial, undertaken under real-world conditions, were that the intervention was not associated with a statistically significant reduction in the prevalence of clinically significant discharge medication error or any discharge medication error, compared to standard care. This is one of the few studies reporting on the evaluation of the implementation of collaborative medication-reconciliation-based interventions, and one of the first reporting on collaborative pharmacist prescribing, in the real-world.^10^ Patients in the intervention group were more likely to receive admission-side pharmaceutical care and pharmacist led medication optimisation activities than standard care. Discharge side pharmaceutical care was rare in both study groups, demonstrating poor intervention fidelity. The findings, consistent with previous evidence, suggested that receipt of pharmacist input and using fewer discharge medicines were protective against error.^7^^,^^19^

### 4.2 Interpretation

Interventions may not produce expected results because of a flawed concept or theory, or due to poor implementation.^10^^,^^25^^,^^26^ Known moderating factors on intervention fidelity include the complexity of the intervention, facilitation strategies, delivery quality and participant responsiveness.^27^ Although intervention pharmacists saw more patients within the cluster than their standard care counterparts, the intervention fidelity, specifically to the discharge element, was poor, reflecting delivery quality. Another possible explanation is that the intervention was implemented in a resource-neutral manner, a possible facilitation strategy. No additional pharmacist whole-time equivalents were employed to deliver the intervention and the pharmacist resource available during the study period was lower than the resource in the pilot study. The intervention pharmacists often had larger patient volumes to be considered for pharmaceutical care provision, particularly the day after a physician or surgeon team was on duty to admit patients from the emergency department (post-take), than ward-based pharmacists, and a larger number of doctors and surgeons to work with than in the pilot study. These can all be considered as moderating factors on fidelity and reflect the challenges with “planning” experienced during implementation of medication reconciliation interventions elsewhere.^28^ Previous research suggests that “patient-level” interventions, such as best possible medication history taking and medication reconciliation by trained personnel, are protective against medication discrepancy.^29^ There is a potential synergistic effect between patient-level and system-level interventions, e.g., the health information technology available to both study groups in the electronic medication reconciliation tool. Our finding that pharmacist input (patient-level) was protective against medication error adds to the existing body of knowledge demonstrating the benefit of pharmacist input in transitional care on medication safety.^1^^,^^30^ However, questions remain about the optimal method to facilitate collaborative pharmaceutical care between physicians/surgeons and pharmacists to the benefit of patient safety in real-world acute hospital contexts.

We formally measured intervention fidelity using process outcome measures, i.e., proportion of patients who received service elements. However, although not within the study protocol, we did record rosters of pharmacists contributing to each cluster. Reflection on this data demonstrates a change in rostering practice that was responsive to workforce availability and resource needs to meet the volume of patients. Although all clusters had input from a pharmacist, as pre-specified, with three years or more post-registration experience and a relevant postgraduate qualification, these pharmacists were frequently aided by a junior colleague who did not fulfil these criteria. This was necessitated in three of the eight clusters to address workload issues, and across multiple clusters to compensate for an unforeseen high volume of patients the morning after the team were “on-take” in the emergency department. This finding demonstrates the iterative managerial responses necessary to manage the spread of an intervention to real-world practice and the benefit of a longer period of data collection to enable the realisation of additional factors that might impact on workload, such as how often a particular team was “on-take” relative to others.

An interaction between the complexity of the intervention and the facilitation strategies employed may also provide explanation: movement from standard care to intervention care involved a steep learning curve for the intervention pharmacists, including the skills associated with collaborative prescribing, enhanced interprofessional working and adapting to each specialty's cultural norms, and it is possible that this distracted from attention to the discharge side components. Such challenges with spread have previously been described.^31^ Again, “education” has been described as an important strategy for medication reconciliation intervention implementation and requires a dedicated programme of stakeholder engagement, repeated and ongoing education and ongoing consultation.^28^ The lead in time of two weeks to facilitate bedding in prior to data collection may have been insufficient to facilitate all the necessary change and learning. Future studies should facilitate longer lead in and measurement time.

Previous evidence from this study site demonstrates the cost-effectiveness of this intervention under controlled conditions and supports the resourcing of pharmacist personnel to deliver the service.^8^ This poses a problem for those who wish to implement such interventions. If a service which has been shown to be effective is partially delivered (e.g., more prevalent admission side service, and little or no discharge side service), there may be a perception that patients receiving the intervention are protected, whereas the reality, as indicated by this study, may differ. The findings demonstrate that the pharmacist input, for both the intervention and standard care groups, was predominantly focussed on the admission side, although a significantly greater proportion of intervention than standard care participants received pharmacist medication history taking and drug chart review. These findings may further highlight the importance of planning and educating when implementing an intervention, and an opportunity for “quality management” by engaging advisory boards who can help monitor and overcome challenges to implementation and support audit and feedback.^8^^,^^10^

Future research should explore the optimal method to deploy scarce clinical pharmacist resource: should more patients receive some clinical pharmacist service, or should fewer patients receive more intensive clinical pharmacist service across the entire inpatient episode? If the latter, further work is needed to implement a reliable prioritisation algorithm in clinical practice.^32^^,^^33^ Future work should also focus on strategies to facilitate wider implementation of discharge side interventions by exploring the challenges to organisational and individual practitioner change, and considering how the collaboration between pharmacists and physicians/surgeons could better prioritise and deliver pharmaceutical care.

### 4.3 Strengths and limitations

This study is one of few exploring the real-world implementation of a complex pharmaceutical care intervention including pharmacist-led medication reconciliation and collaborative pharmacist prescribing. The study findings should be contextualised within its potential limitations, and the risk of bias identified as ‘low’ for two domains, and ‘some concerns’ for three domains (Supplementary material). The investigator collecting the data and the personnel delivering the intervention were not blinded to study group (i.e., there was no allocation concealment), potentially contributing to selection and performance bias, respectively. Individual patient recruitment has been identified as a source of bias in SW-CRTs, although it is an area which has not yet been the subject of much research.^14^ The possibility of contamination introduced by cross-over of junior doctors participating between study groups cannot be out-ruled. The pharmacists delivering the “standard” and “intervention” care were essentially the same pool of pharmacists, with “intervention” pharmacists who were not yet deployed delivering standard care in earlier phases and junior pharmacists assisting “intervention” pharmacists for workload reasons.

The sample size calculation was based on a parallel cluster design rather than being specific to SW-CRTs. Formal methods for sample size and power calculation for SW-CRTs have been described in the statistical literature, although little guidance was available at the time of study design in 2014. This is reflected in the results of a systematic review which identified shortcomings in the methodological standards of many published SW-CRT.^34^

The stepped wedge study design presented a pragmatic choice for this study because random allocation of patients would have been difficult and otherwise a weaker, non-randomised study design would have been needed. ^14^^,^^35^ While the ICC values were found to be minimal or not definable, clusters were necessary to protect the integrity of the intervention and to prevent contamination between groups. Clusters followed the implementation schedule at designated six-weekly intervals according to randomisation, with one exception where staffing shortages resulted in the postponement by one week of initiation of the intervention in the final two clusters. Within-cluster analyses were not undertaken because of the risk of bias due to confounding of the treatment effects over time.^13^ Temporal effects are possible within SW-CRTs and can be modelled.^36^ Timepoint, in the form of study phase, was adjusted for in the regression analyses and was not statistically significant in the ITT analysis. Therefore, the generalisability of the trial findings is robust within the recruited clusters, but likely limited beyond that and therefore should be treated with caution in supporting similar interventions in other settings.

The process evaluation was limited to assessment of intervention fidelity to determine the extent of implementation. A more complete process evaluation could investigate how and why the intervention was delivered, not just what parts were delivered, and consider the contextual factors, such as the training and support provided to pharmacists delivering the intervention, management, workforce and other resources, and communication aspects. The external features which act as barriers or facilitators to implementation or outcomes, and the mechanisms of impact should also be explored through dedicated quantitative and qualitative analyses. ^10^^,^^26^

No patients were involved in the research, an oversight which potentially limits the rigour of the work and negatively impacted on implementation success.^10^ Additionally, the perception and experience of patients receiving care were not captured, a critical oversight that should be addressed in future transitional medication safety research to ensure the research question, data collection and outcome measures are validated by service users. Finally, the study was not registered as a clinical trial.

## 5 Conclusion

Under real-world conditions, the effect of this collaborative model of pharmaceutical care including medication reconciliation and collaborative prescribing did not differ from standard care in protecting against clinically significant, or indeed any, medication error. Future research should employ an implementation science framework to better understand how the intervention can be spread to achieve positive and sustained patient outcomes.

DME = discharge medication error, OR = odds ratio.

## Funding sources

This work was supported by a research grant from The Meath Foundation and the Tallaght Hospital Pharmacy Education and Research Fund.

## CRediT authorship contribution statement

**Gráinne Kirwan:** Writing – review & editing, Project administration, Methodology, Formal analysis, Data curation, Conceptualization. **Ann Allen:** Project administration. **Evelyn Deasy:** Writing – review & editing, Supervision, Project administration, Funding acquisition, Conceptualization. **Tim Delaney:** Writing – review & editing, Funding acquisition, Conceptualization. **Jennifer Hayde:** Writing – review & editing, Project administration, Conceptualization. **Ciara McManamly:** Writing – review & editing, Project administration, Conceptualization. **Catherine Wall:** Writing – review & editing, Project administration, Conceptualization. **John O'Byrne:** Writing – review & editing, Project administration, Funding acquisition, Conceptualization. **Tamasine Grimes:** Conceptualization, Writing – review & editing, Writing – original draft, Supervision, Project administration, Methodology, Funding acquisition, Formal analysis.

## Declaration of competing interest

The authors declare that they have no known competing financial interests or personal relationships that could have appeared to influence the work reported in this paper.

## Glossary

SW-CRT: stepped wedge cluster randomised trial
PAML: pre-admission medication list
ICC: intraclass correlation coefficient
ITT: intention-to-treat
PP: per-protocol
GLMM: generalised linear mixed model

## Appendix A Supplementary data

Supplementary material Image 1
