# Supplementary material for: The impact of collaborative pharmaceutical care on hospital discharge medication error prevalence: A stepped-wedge cluster randomised trial
Source: Explor Res Clin Soc Pharm. 2025 Jul 31;20:100638. doi: 10.1016/j.rcsop.2025.100638 (PMC12354959; doi:10.1016/j.rcsop.2025.100638)
Supplement: Supplementary file 1 — Supplementary material [file mmc1.docx]

# Supplemental material

## Comparison of baseline characteristics between study groups

**Table S1. Comparison of baseline demographic and clinical characteristics of the included patient population by arm**

| **Characteristic** | **Standard care (n = 213)** | **PACT (n = 251)** | **p-value, coefficient** |
| --- | --- | --- | --- |
| Gender, female, n (%) | 104 (49%) | 114 (45%) | 0.515, 0.537^p^ |
| Age, years, median (IQR) | 69 (61-78) | 68 (59-80) | 0.852, 26462.50^m^ |
| Urgency of presentation, n (%) |  |  | 0.261, 2.684^p^ |
| *Emergency* | 183 (85.9) | 225 (89.6) |  |
| *Elective* | 30 (14.1) | 25 (10.0) |  |
| *Unknown* | 0 (0.0) | 1 (0.4) |  |
| Number of pre-admission medications, median (IQR) | 9 (6.5-11.0) | 9 (7-12) | 0.341, 25369.50^m^ |
| Comorbidity index*, median (IQR) | 1 (0-2) | 1 (0-2) | 0.980, 26696.50^m^ |

*Charlson Comorbity Index adapted by Quan et al (2011), ^m^ = Mann Whitney U, ^p^ = Pearson’s chi-square

## Intention-to-treat (ITT) analysis of the primary outcome measure

**Table S2. Independent variables and associated odds ratio (OR) within the adjusted logistic regression model for the ITT analysis**

| **Variable** | **Beta** | **OR*** | **95% CI for OR**  ***Lower Upper*** | | **p-value** |
| --- | --- | --- | --- | --- | --- |
| Pharmacist input present | -1.241 | 0.289 | 0.168 | 0.497 | <0.001 |
| Study phase† |  |  |  |  |  |
| *Phase 2* | -0.660 | 0.517 | 0.195 | 1.372 | NS |
| *Phase 3* | -0.543 | 0.581 | 0.257 | 1.312 | NS |
| *Phase 4* | -0.774 | 0.461 | 0.183 | 1.165 | NS |
| *Phase 5* | -0.710 | 0.492 | 0.163 | 1.480 | NS |
| Age | -0.004 | 0.996 | 0.980 | 1.013 | NS |
| Gender | 0.085 | 1.088 | 0.647 | 1.830 | NS |
| Number of medicines | 0.078 | 1.082 | 1.022 | 1.144 | <0.05 |

* OR is equal to Exp (Beta)

†Reference is Phase 1

NS = Not statistically significant
